# DNA-based occurrence dataset on peatland fungal communities studied by metabarcoding in north-western Siberia

**DOI:** 10.3897/BDJ.12.e119851

**Published:** 2024-03-27

**Authors:** Nina Filippova, Elena Zvyagina, Elena A. Rudykina, Tagir F. Ishmanov, Ilya V. Filippov, Tatiana M. Bulyonkova, Alevtina S. Dobrynina

**Affiliations:** 1 Yugra State University, Khanty-Mansiysk, Russia Yugra State University Khanty-Mansiysk Russia; 2 Moscow State University, Moscow, Russia Moscow State University Moscow Russia; 3 Yugra State University, Novosibirsk, Russia Yugra State University Novosibirsk Russia

**Keywords:** peat, histosols, sphagnum, fungi, environmental DNA, eDNA, GBIF

## Abstract

**Background:**

The paper represents the first DNA-based occurrence dataset on peatland fungal communities published for north-western Siberia, the first for Russia and complements several existing datasets on metabarcoding of peat soils globally.

**New information:**

The aim of the present publication is to describe the first DNA-based occurrence dataset on fungal communities in peat soils and other substrates studied by the eDNA approach in the Mukhrino raised bog, located in a large paludified area of north-western Siberia. A comparison of the species diversity of larger fungi identified by the conventional approach and by eDNA showed a high proportion of shared taxa. Other groups (mainly Ascomycota), described by metabarcoding, revealed high diversity compared with conventional observation. Overall, the species richness identified in one peatland locality (the Mukhrino Bog) was comparable in number of species to the global estimation of fungal diversity in peatlands, previously reported in literature.

## Introduction

Peatlands are a special ecosystem type that forms in humid conditions when large masses of organic carbon accumulate and form the peat layer in anoxic conditions (Wider and Vitt 2006). This layer is withdrawn from the carbon cycle and deposited; peatlands have value for human applications (like fuel or fertilisers) and as a means to combat climate change. Globally, peatlands cover up to 3% of the terrestrial surface, but store about 30% of the world's terrestrial soil carbon (Wieder et al. 2006). While peatlands are found almost in every country, a third of all peatlands are concentrated in a few large continuous peatland areas (Kirpotin et al. 2021). The object of this study is peat soils and peatlands in north-western Siberia, where the whole area is highly paludified, with up to 50–70% of the land covered by peat soils that have developed here since the end of the Last Glacial Period (Kremenetski et al. 2003).

The study of the fungal diversity of peatlands globally started over a century ago and was described in a series of reviews (Rydin and Jeglum 2006, Thormann 2006, Artz 2013) and in our previous publication (Filippova et al. 2023d). Studies of fungal diversity in different types of peatlands were carried out in a variety of paludified regions in different countries. Different approaches were chosen, most often a microbiological approach using cultivation techniques and a direct observation approach of collecting or counting the fruiting structures of larger fungi. As estimated globally, there are about 600 species of fungi described in peatlands and included in the first checklist of peatland fungi (Thormann and Rice 2007), but about 1500 species presently, according to our accumulated literature-based peatland fungi dataset (Filippova and Rudykina 2023).

The metabarcoding of fungi has greatly improved the global estimate of fungal diversity and provided valuable insights into the ecological composition of fungal communities in various ecosystems (Hibbett et al. 2009, Větrovský et al. 2020, Tedersoo et al. 2022). In peatland ecosystems, the method was employed in a few published works to date: Jackson et al. (2008), Elliott et al. (2015), Garcés-Pastor et al. (2019), Vašutová et al. (2021). However, the diversity and structure of fungal communities in peatlands in north-western Siberia were not described by this modern approach, leaving a large gap on a global map of eDNA data on fungi in this area (Větrovský et al. 2020).

This dataset complements a series of published open datasets on fungal communities in north-western Siberia and globally, complementing the complex approach to the research of peat soils (conventional observation, barcoding and metabarcoding):

1. A sampling-event dataset was published in 2020 and has been updated yearly, currently representing 10 years of plot-based surveys of larger fungi in the Mukhrino Bog (Filippova and Lapshina 2019, Filippova et al. 2023c);
2. An occurrence dataset with DNA-derived extension was published recently following the barcoding of the specimens collection from the Mukhrino Raised Bog (Filippova et al. 2023a, Filippova et al. 2023d);
3. A literature-based occurrence dataset (citations of published sources) was initiated and will be updated regularly as new research on peatland fungi worldwide emerges (Filippova and Rudykina 2023).

The standardised approach for data storage of metabarcoding results in general and fungal metabarcoding specifically has been developed in recent years (Nilsson et al. 2018a, Nilsson et al. 2018b, Martorelli et al. 2020, Tedersoo et al. 2022). Fungal molecular taxonomic units are being accumulated and processed on a fungi-specific web-based platform UNITE and integrated with the taxonomic backbone of the Global Biodiversity Information Facility (Nilsson et al. 2018b). As the accumulation of DNA-based occurrences of species and integration of the data into biodiversity data platforms is becoming more relevant, GBIF provides new instruments and guidelines to publish and discover such data (Abarenkov et al. 2023).

The study of the peatland fungal community in the vicinity of Mukhrino field station (the middle taiga zone of north-western Siberia) has been carried out for over a decade. The permanent plot-based monitoring of the fruiting dynamics of larger fungi was initiated in 2014 and continues to date with biweekly counts on 5 m^2^ circular plots on a total area of 1315 m^2^ (Filippova and Lapshina 2019). The accumulated specimen collection of larger fungi was studied and verified recently by a molecular approach and revealed a total of 95 species (based on morphological and sequence identification), including several potentially new species (Filippova et al. 2023d). A collection of plant leaf saptrotrophs was created, covering several of the most common bog plants and included a quantitative study of fungal saptotrophs of *Andromedapolifolia* L. leaf litter (Filippova 2012, Filippova 2015, Filippova and Thormann 2015). The approximate species diversity of leaf saprotrophs revealed about 150 species, but needs further revision by a molecular barcoding approach. The wood decay community in the same raised bog revealed by direct observation of fruiting structures yielded about 50 species from the Ascomycota and Basidiomycota (Filippova and Zmitrovich 2013). Some research was done on the fungal diversity of yeasts (Kachalkin 2010) and terrestrial lichens (Lapshina and Koneva 2010) of the area. Overall, the checklist of taxonomic diversity of fungi from the raised bog Mukhrino contains around 300 taxa, although the majority of them require further confirmation using a molecular identification approach.

To supplement direct observation of fruitbodies with an environmental DNA approach, we completed a series of samplings of common substrates in the same locality in the Mukhrino Bog. Four major substrates were subjected to metabarcoding analysis: peat (from the surface layer to a depth of about 3 m), leaf litter of six bog plants, wood (represented by standardised wooden dowels) and mycorrhizal roots of two bog-dwelling trees. Metabarcoding of the ITS2 region (Illumina MiSeq platform) revealed about 1200 OTUs and 800 Linnean taxa. The community analysis of different substrates, based on metabarcoding results, showed significant differences between all four substrates; a high difference between two different bog habitats (hummocks and hollows); a significant difference between all litter types of bog plants; and an insignificant difference between the roots of two bog pine species. The results also showed a high influence of season on community composition (from the beginning to the end of summer) and a high influence of peat depth parameter for the community of peat substrate.

The taxonomic diversity revealed by the eDNA approach was compared with earlier results at three levels: 1) with the global literature-based checklist of fungi in peatlands based on a literature dataset; 2) with the accumulated checklist of fungi in the Mukhrino Bog, based on an earlier conventional approach; 3) to verify both approaches at more strict limits, we made the comparison of larger fungi (Agaricomycetes) revealed most thoroughly in the Mukhrino Bog by a ten-year direct observation period with the same group revealed by eDNA analysis.

## Sampling methods

### Study extent

**Figure 1.**
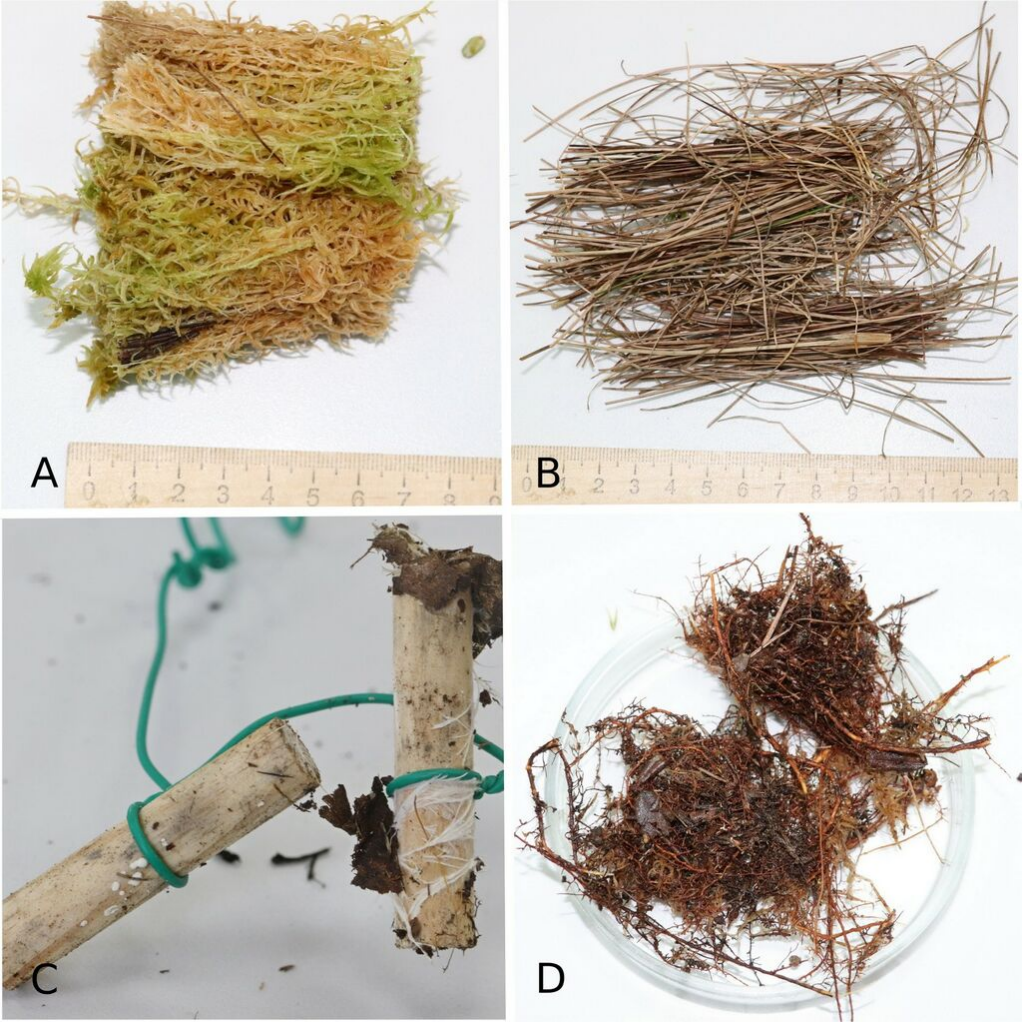
Four substrates studied by metabarcoding analyses: A – peat (from the surface layer to about 3 m depth, the surface sample from 0-5 cm depth shown as example); B – plant litter (*Eriophorumvaginatum* L. dead leaves shown as example); C – wood (standardised wooden dowels); D – mycorrhizal roots of *P.sylvestris* and *P.sibirica*.

**Figure 2.**
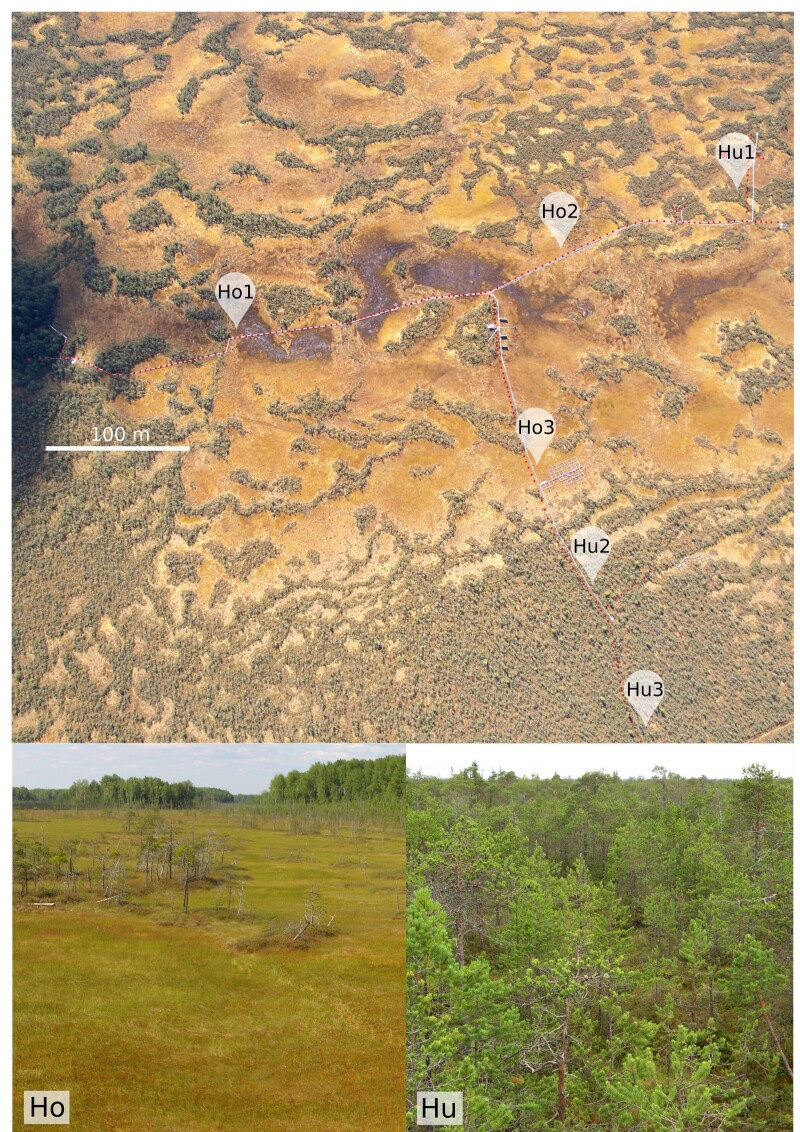
Layout of the Mukhrino field station infrastructure and position of plots (Hu – hummock, Ho – hollow) where metabarcoding samples were extracted (plots used only for peat and leaf litter samples); red dots mark the position of circular 5 m^2^ plots for monitoring larger fungi; lower right insert: overview of two habitats (hummock and hollow).

**Figure 3.**
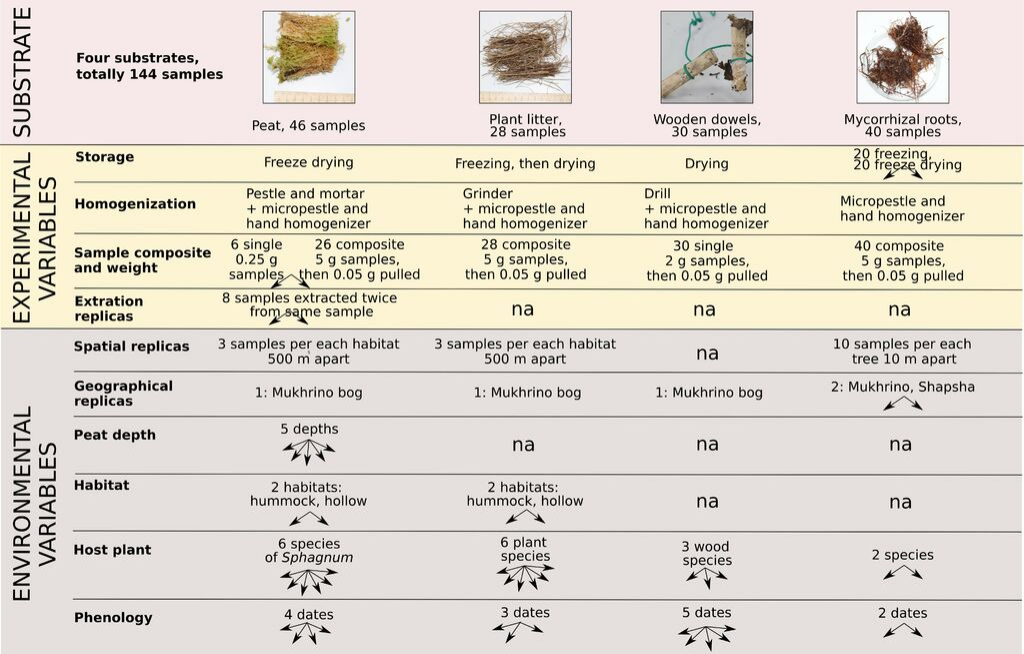
Experiment design for metabarcoding analysis of fungal communites in the Mukhrino Bog (including four different substrate types, experimental and environmental variables).

In order to study the fungal community of raised bogs, four major substrates were subjected to metabarcoding analysis: peat (from the surface layer to about 3 m depth), plant litter (6 plant species), wood (standardised wooden dowels) and mycorrhizal roots (*Pinussylvestris* L., *P.sibirica* Du Tour) (Fig. 1). Six plots were located alongside the walking board of the Mukhrino field station research polygon in two habitats: treed Pine-dwarf-shrubs-*Sphagnum* bogs (hummock, Hu) and graminoid-*Sphagnum* hollows (hollow, Ho) (Fig. 2). For each of the substrate groups, we designed the experiment to cover spatial and temporal variability, substrate features and methodological questions related to sample size, storage and homogenisation approaches (see metadata table with environmental and experimental parameters of all 144 samples (Fig. 3 and Filippova (2023)).

### Sampling description

All field operations were made wearing gloves and the instruments (knife, scissors and tweezers when necessary) were sterilised between samples with bleach and alcohol according to recommendations (Tedersoo et al. 2022). Samples were wrapped in sterilised aluminium bags and labelled with permanent markers. Bags with samples were put in a cooling bag with a cooling agent immediately after sampling and transported to the laboratory to be frozen at −22°C within a few hours. All substrates, except wood, were frozen at −20°C in a refrigerator to be extracted and processed 2 months later. Wooden dowels were wrapped in paper bags, dried in a drying cabinet at 40°C for 24 hours and stored in a dry stage before extraction according to recommendations (Shumskaya et al. 2023).

### Step description

**Sampling of peat and eDNA extraction protocol**

To study the fungal community of peat, six plots were located in two habitats: hummocks and hollows (Fig. 2). Each plot was sampled regularly in June, July, August and September. Several shoots of dead *Sphagnum* L. were collected from 10 points 5 m apart within each plot to create a composite sample with a field weight of approximately 5 g. To test the efficiency of the sampling approach, an experimental sampling from a single point (0.05 g, several shoots) was made in June and was later compared with the composite sample in the same plot. All samples of peat were lyophilised and homogenised manually:

1. using a sterilised pestle and a mortar for 5 g composite samples;
2. using sterilised micropestles for single 0.05 g samples. From each composite sample, about 0.05 g (0.3 ml) of peat powder was transferred to a 1.5 ml Eppendorf tube. To test the homogeneity of the composite sample, two replicas of peat powder were taken from 8 samples (extraction replicas) and then compared.

**Figure 4.**
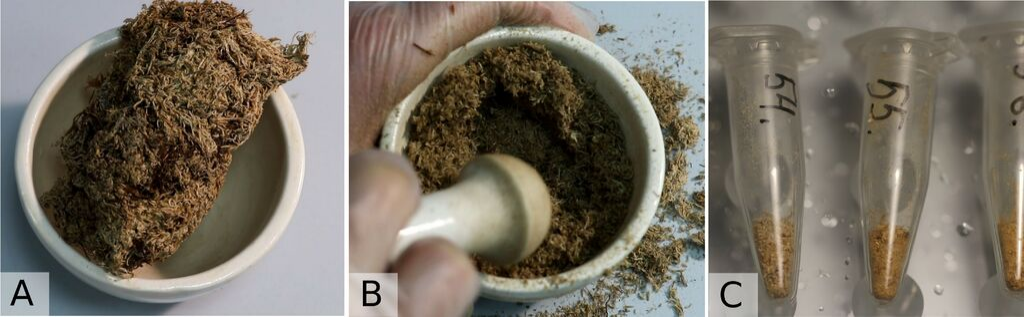
Sample preparation of peat: A – freeze-dried sample of *Sphagnumfuscum* (Schimp.) H.Klinggr. (about 5 g field weight), B – homogenisation using a pestle and a mortar, C – approximately 0.05 g of peat powder pooled from each sample (to be further processed according to manufacturer instructions).

All samples were soaked in 400 µl of lysis buffer overnight, then homogenised using a micro-tube homogeniser with glass beads according to manufacturer instructions (total DNA extraction soil kit, SileksMagNA) (Fig. 4). The general sampling depth was about 2–5 cm below the *Sphagnum* surface. Additional sampling at different depths was done in August, where the samples were collected in two plots at four different depths (0–2 cm, 5–7 cm, 10–15 cm and 25–30 cm below the surface). The sampling at deeper peat horizons up to the mineral layer (about 3 m depth) was done in the summer of 2023 to study the potential activity of the community in the deeper catotelm layers, but these samples will be analysed later and will not be discussed in the present publication.

This experiment design resulted in a total of 46 samples with the following environmental variables for analysis: habitat (2 types), species of *Sphagnum* (6 species), peat depth (4 depths) and seasonal variation (4 dates); and experimental variables to test: the efficiency of sampling approach (composite 5 g vs. single 0.05 g samples); the efficiency of sample homogenisation and extraction replicas (Fig. 3).

**Sampling of plant litter and eDNA extraction**

The community of fungal saprotrophs of the six common plant species was studied by collecting their leaf litter: *Rhododendrongroenlandicum* (Oeder) Kron & Judd, *Chamaedaphnecalyculata* (L.) Moench, *Rubuschamaemorus* L. (in hummock habitats), *Andromedapolifolia* L., *Eriophorumvaginatum* L. and *Scheuchzeriapalustris* L. (in hollow habitats). The litter was picked randomly from the surface of *Sphagnum* over an area of approximately 10 m^2^ in each plot. Sampling was performed in the same plots and on the same dates as the peat substrate (see above). A total of 5 g of field weight substrate of each plant was collected three times per season (June, July and September), totalling 28 samples.

**Figure 5.**
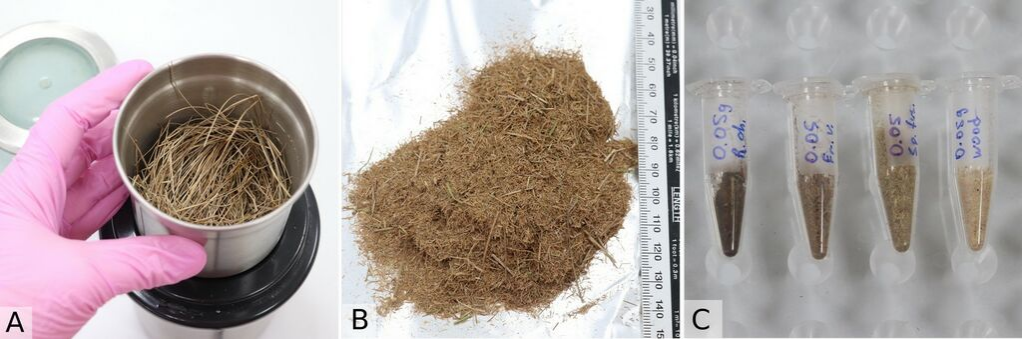
Sample preparation of litter: A – dried sample of *E.vaginatum* (about 5 g of field weight), B – homogenisation using a grinder, C – about 0.05 g of litter powder (to be further proccessed according to manufacturer instructions).

All samples were packed in sterile paper bags and dried in a dehydrator at 40°C. Each sample was then ground in a coffee grinder (all parts were sterilised between the samples) in order to break down hard plant material and homogenise the composite sample. From each composite sample, about 0.05 g of plant powder was transferred to a 1.5 ml Eppendorf tube, soaked and homogenised with a lysis buffer as above (Fig. 5).

**Sampling of mycorrhizal roots and eDNA extraction**

**Figure 6.**
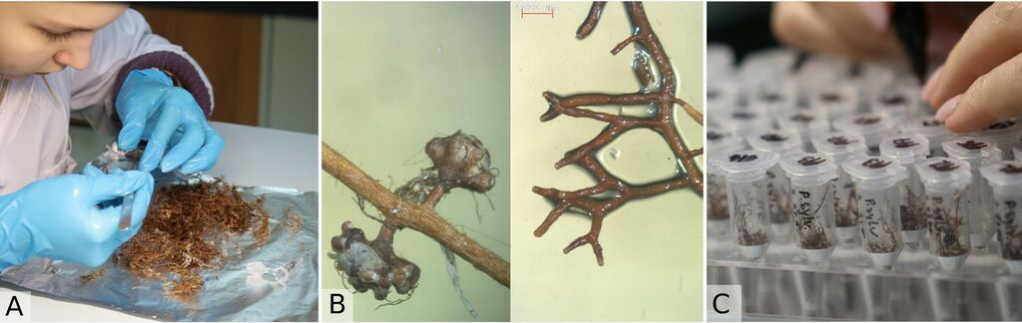
Sample preparation of mycorrhizal roots: A. cleaning the roots from fine debris; B. example of cleaned mycorrhizal roots under dissecting microscope; C. collecting about 0.5 ml of fine roots in each Eppendorf tube.

To study the mycorrhizal community of bog trees, we collected the ectomycorrhizal roots of two common bog-dwelling trees: *P.sylvestris* and *P.sibirica*. The roots were collected in two localities («Mukhrino» and «Shapsha», located about 30 km from each other across the Ob-Irtysh River confluence) for geographical variability. In each locality, 5 to 10 trees growing about 10 m apart were marked for the following root extraction and dendrochronological boring. Sampling was done twice a year at the beginning and at the end of the vegetation season (June and September), producing a total of 40 root samples. About 30 g of fine roots were extracted from samples taken about 20–30 cm apart in several sites around each tree trunk. The samples were additionally cleaned from fine debris in the laboratory; the cleaned roots were collected in Eppendorf tubes (about 0.5 ml volume) and frozen (Fig. 6). The roots were homogenised by two different approaches to compare their final performance. The first group of samples was lyophilised and then homogenised using a micro-tube homogeniser to create dry fine powder. The second group of samples was homogenised directly (without lyophilisation) using a micro-tube homogeniser and glass beds accordingly.

**Sampling of wood and eDNA extraction**

**Figure 7.**
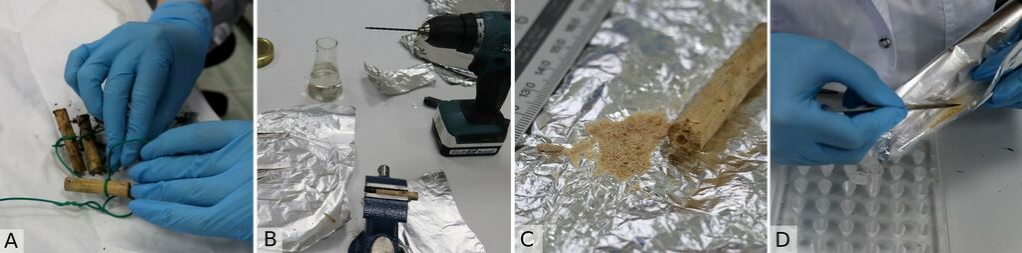
Sample preparation of wooden dowels: A. cleaning dowels from outer debris; B. drilling the interior of each dowel; C. the drilled-out dowel and sawdust; D. collecting sawdust in plastic tubes.

To study the total DNA of the dead wood community, we used an approach of standardised substrates (Shumskaya et al. 2023) developed to describe the community in the early stages of wood decay. Sterilised wooden dowels of three tree species (pine, larch and birch) were buried in the upper peat surface in hummock habitats and were extracted at two-week intervals throughout the season. The collected dowels were wrapped in sterile bags and dried at 40°C for a day. A total of 30 wood dowels were extracted by the end of the first season. The homogenisation of wood substrates was done according to the following: the interior of each dowel was drilled by a 2 mm fire-sterilised drill bit and the sawdust was collected into sterile plastic centrifuge tubes (Fig. 7). Further extraction was done as above, by addition of 40 µl of lysis buffer, soaking and homogenising with glass beads according to the instructions of the SileksMagNA kit.

**DNA detection, library preparation, PCR and sequencing**

A total of 144 samples of environmental DNA, extracted from four substrates, were obtained and stored at −20°C until being processed. The samples of extracted DNA were outsourced for processing by an independent company (Evrogen, Moscow). The quality of the obtained metagenomic DNA was checked by electrophoresis on an agarose gel. Quantification was carried out by measuring the concentration of DNA by Qubit 2, using the dsDNA HS reagent kit (ThermoFisher Scientific). The preparation of libraries for sequencing was carried out in accordance with the protocol described in 16S Metagenomic Sequencing Library Preparation (Part #15044223 Rev. B; Illumina). Amplification of ITS variable regions was carried out using primers: fITS7: 5'-GTGARTCATCGAATCTTTG-3' and ITS4: 5'-TCCTCCGCTTATTGATATGC-3' (White et al. 1990, Ihrmark et al. 2012). After obtaining the amplicons, the libraries were purified and pooled equimolarly with the SequalPrep™ Normalization Plate Kit (ThermoFisher, Cat #A10510-01). Quality control of the libraries was carried out using the Fragment Analyzer and quantitative analysis was carried out with qPCR. The library was sequenced on Illumina MiSeq (length of reads – 300 bp on both side fragments) using MiSeq Reagent Kit v.3 (600 cycles). FASTQ files were obtained using bcl2fastq v.2.17.1.14 Conversion Software (Illumina). The PhiX phage library was used to control sequencing parameters. Most of the readings related to phage DNA were removed during demultiplexing.

**Raw data storage.** The raw reads (FastQ archives and a metadata table) were uploaded to NCBI Sequence Reads (bioproject accession number PRJNA1007262).

**Sequence processing and bioinformatics pipeline**

The obtained sequences were processed using QIIME2 (Quantitative Insights Into Microbial Ecology 2, version 2023.9).

The pipeline of sequence analysis is applied as follows:

1. Indexes were removed using trim-paired (QIIME cutadapt trim-paired);
2. Forward and backward reads were merged using merge-pairs (QIIME vsearch merge-pairs);
3. Quality filtering done using q-score (QIIME quality-filter q-score);
4. Dereplication made using dereplicate-sequences (QIIME vsearch dereplicate-sequences);
5. Internal de-novo clustering with an identity parameter of 99% (QIIME vsearch cluster-features-de-novo);
6. Clustering based on the UNITE database (version 9.0 16 October 2022) using cluster-features-closed-reference with 97% identity parameter (QIIME vsearch cluster-features-closed-reference);
7. Chimeras removed using uchime-ref (QIIME vsearch uchime-ref);
8. Classification classify-sklearn (QIIME feature-classifier classify-sklearn) on a classifier that was trained using the naive Bayes classifier algorithm (QIIME feature-classifier fit-classifier-naive-bayes);

**Curated sequence classification**

To make the automatic classification plausability check, manual curated sequence classification was performed for the most locally studied group of larger agaricoid fungi:

1. All sequences were filtrated by the following taxa to select the group: order Agaricales, Boletales and Russulales; families: Agaricaceae, Auriscalpiaceae, Boletaceae, Clavariaceae, Cortinariaceae, Crepidotaceae, Entolomataceae, Hygrophoraceae, Inocybaceae, Lycoperdaceae, Lyophyllaceae, Mycenaceae, Omphalotaceae, Paxillaceae, Physalacriaceae, Pluteaceae, Psathyrellaceae, Russulaceae, Strophariaceae, Suillaceae, Thelephoraceae, Tricholomataceae; and some selected genera from other families.
2. The NCBI BLAST search for all OTUs was performed to find the nearest sequences from a type specimen, an authentic specimen or a voucher sequence specimen from the YSU-F collection with a percentage identity conventionally accepted (for example, 99% for Cortinarius, Liimatainen et al. (2020)). In case no type or authentic specimen existed in NCBI, any other reliable sequence was chosen.
3. The sequences of each group were aligned with the nearest sequence of a type, an authentic specimen and a voucher specimen, trimmed for maximum overlap and the ITS2 region with the number of nucleotides conventional for this group was left.
4. Names were assigned, based on similarity to the closest taxon. Most of the sequences had 99–100% percentage similarity and were assigned to species level. Sequences with a much lower threshold (98% and less) were left at the genus level. These taxa were assigned consecutive numbers (shared by metabarcoding sequences and voucher sequences from the YSU-F collection (Filippova et al. 2023d).

## Geographic coverage

### Description

The study sites are the Mukhrino field station and the Mukhrino Bog, which are located in the middle taiga zone of Western Siberia, near the regional capital city of Khanty-Mansiysk (60.89°N, 68.68°E). The Mukhrino Bog is an ombrotrophic landscape entity covering an area of about 10 by 15 km, located along the northern edge of a larger paludified area, the Konda Lowlands (Russian Кондинская низменность), on the left terrace of the Irtysh River close to its confluence with the Ob'. The vegetation of the raised bog is represented by the typical ombrotrophic or oligo-mesotrophic communities from the vegetation classes *Scheuchzerio-Cariceteanigrae*, *Oxycocco*-*Sphagnetea* and *Vaccinio*-*Piceetea*. Two major vegetation types dominate: tree Scots pine – dwarf shrubs – *Sphagnum* hummocks dominated by *Pinussylvestris*, *Chamaedaphnecalyculata*, *Rhododendrongroenlandicum*, *Rubuschamaemorus* and *Sphagnumfuscum*) and open graminoid-Sphagnum hollows (dominated by *Scheuchzeriapalustris*, *Carexlimosa* L., *Eriophorumrusseolum* Fr., *Vacciniumoxycoccos* L. and *Sphagnumbalticum* (Russow) C.E.O.Jensen).

### Coordinates

60.89151 and 61.06549 Latitude; 68.67719 and 69.45882 Longitude.

## Taxonomic coverage

### Description

The sequence analysis revealed a total of 1259 OTUs classified into 471 species, 423 genera, 223 families, 86 orders, 30 classes, seven phyla and one kingdom at a 99% similarity level. About 42% of taxa were identified at the species level, 21% at the genus level and the rest at higher taxonomic levels (Table 1).

**Figure 8.**
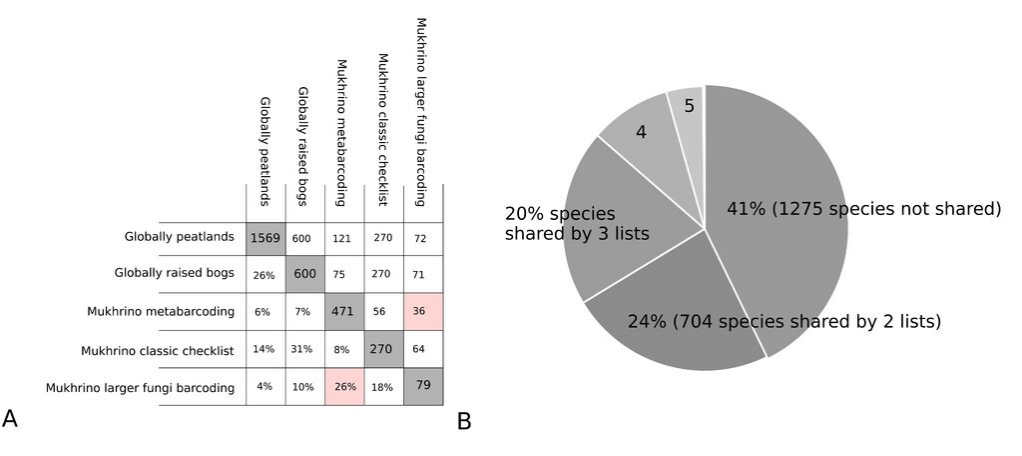
Number and percentage of species shared by different checklists (global literature-based dataset, metabarcoding results in the Mukhrino Bog and conventional approach in the Mukhrino Bog): A. a matrix of percentage (lower part) and number of shared species (upper part) between each two lists; B. a diagram showing percentage of species shared by several checklists.

To compare the revealed taxonomic diversity with earlier published results, we used several checklists of fungi in peatlands:

1. A global checklist of fungi from peatlands, compiled from a literature-based occurrence dataset (Filippova and Rudykina 2023). The taxonomic structure of fungal diversity represented by the dataset (after synonimisation using the GBIF species matching tool) includes three kingdoms (Fungi, Chromista and Protozoa), seven phyla, 27 classes, 87 orders, 239 families, 616 genera and about 1500 species. The larger fungi represent about 80% of occurrences and 1100 species, while microfungi only represent about 400 species. The species list of fungi revealed by metabarcoding in Mukhrino shared only 121 species (6%) with the global checklist.
2. A checklist of fungi from raised bogs, selected from the previous dataset, based on habitat descriptions in the original publication. The resulting checklist contains about 600 species found in specific raised bog habitats. The similarity percentage was 7% (75 shared species).
3. A checklist of fungi collected by conventional approach (direct observation) or through cultivation in the Mukhrino Bog was created, based on a selection of literature sources published specifically about the Mukhrino Bog (a total of about 270 species). Despite the same locality of sampling, the percentage similarity remains low: 8% (56 shared species).
4. For reliability purposes, we limited both lists to one large monophyletic group of larger fungi (Agaricales), which has been most fully studied by conventional approaches in the Mukhrino Bog and confirmed by barcoding of voucher specimens. This resulted in the highest similarity between the two approaches (metabarcoding vs. conventional collection): 26% (36 species) shared, while 59 were unique for conventional collection and 44 revealed only by eDNA (Fig. 8)

**Curated sequence classification results**

The curated sequence classification showed quite significant differences when compared at the species level. Both classifications showed 100% similarity at the class, order, family and genus taxonomical ranks. However, at the species level, 23% species (27 from 118) were assigned different names as a result of curated classification: nine species were re-identified as other species, 14 taxa improved identification to species level and four species were reduced to genus level (Table 2).

### Taxa included

**Table:** 

Rank	Scientific Name	
kingdom	Fungi	

## Temporal coverage

### Notes

2022-06-01 through 2022-09-01

## Usage licence

### Usage licence

Other

### IP rights notes

This work is licensed under a Creative Commons Attribution (CC-BY) 4.0 License.

## Data resources

### Data package title

DNA-based occurrence dataset of peatland fungal communities studied by metabarcoding in north-western Siberia

### Resource link

https://www.gbif.org/dataset/aa9fabb1-e7c0-4265-aa7b-8e85bf73f3dd

### Alternative identifiers

http://ipt.ugrasu.ru:8080/resource?r=bogfunmeta

### Number of data sets

2

### Data set 1.

#### Data set name

DNA-based occurrence dataset on peatland fungal communities studied by metabarcoding in north-western Siberia

#### Data format

Darwin Core

#### Character set

UTF-8

#### Download URL

http://ipt.ugrasu.ru:8080/archive.do?r=bogfunmeta

#### Description

The dataset representing DNA-based occurrences published in GBIF as an occurrence dataset with the DNA-derived extension table based on guidelines (Abarenkov et al. 2023). The dataset contains two tables. The first table (Occurrence Core) has 20 fields to describe features of samples and observed taxonomic occurrences with their abundances (number of reads); the table contains a total of 9,749 occurrences. The related DNA-derived data table contains sequences linked to each occurrence with their metadata (Filippova et al. 2023b).

**Data set 1.** 

Column label	Column description
occurrenceID (Occurrence core)	A unique identifier for the occurrence.
scientificName (Occurrence core)	An OTU identifier from UNITE.
scientificNameAuthorship (Occurrence core)	The authorship information for the scientificName.
Kingdom (Occurrence core)	The full scientific name of the kingdom in which the taxon is classified.
Phylum (Occurrence core)	The full scientific name of the phylum in which the taxon is classified.
Class (Occurrence core)	The full scientific name of the class in which the taxon is classified.
Order (Occurrence core)	The full scientific name of the order in which the taxon is classified.
Family (Occurrence core)	The full scientific name of the family in which the taxon is classified.
Genus (Occurrence core)	The full scientific name of the genus in which the taxon is classified.
specificEpithet (Occurrence core)	The name of the first or species epithet of scientificName.
eventID (Occurrence core)	An identifier for the set of information associated with an Event (sample number).
organismQuantity (Occurrence core)	Number of reads of this OTU in this sample.
organismQuantityType (Occurrence core)	"DNA sequence reads".
habitat (Occurrence core)	A category or description of the habitat in which the dwc:Event occurred ("Treed pine-dwarfshrubs-sphagnum ombrotrophic raised bog" or "Graminoid-sphagnum lawn of ombrotrophic raised bog").
sampleSizeValue (Occurrence core)	Total number of reads in the sample.
sampleSizeUnite (Occurrence core)	"DNA sequence reads".
decimalLatitude (Occurrence core)	The geographic latitude where the dwc:Event occurred (exact locality of the sample collection).
decimalLongitude (Occurrence core)	The geographic longitude where the dwc:Event occurred (exact locality of the sample collection).
eventRemarks (Occurrence core)	Substrate type ("Mycorrhizal roots", "Peat", "Wooden dowels" and "Plant litter") and peat depth.
associatedTaxa (Occurrence core)	A name of plant from which the sample was collected (for example, "host":"Pinussylvestris").
eventDate (Occurrence core)	Date when the sampling of substrate was made.
country (Occurrence core)	A name of the country where the sampling occurred ("Russia").
countryCode (Occurrence core)	The standard code for the country ("RU").
geodeticDatum (Occurrence core)	The geodetic datum ("WGS84").
coordinateUncertaintyInMetres (Occurrence core)	The coordinate uncertanty (all coordinates taken with GPS with uncertainty about 30 m).
ID (DNA-derived extension)	A unique identifier for the occurrence refers to the occurrence table (occurrenceID).
DNA_sequence (DNA-derived extension)	The DNA sequence (OTU).
sop (DNA-derived extension)	Standard operating procedures used in assembly and/or taxonomic annotation of reads.
target_gene (DNA-derived extension)	Targetted gene or marker name for marker-based studies (ITS).
target_subfragment (DNA-derived extension)	Name of subfragment of a gene (ITS2).
pcr_primer_forward (DNA-derived extension)	Forward PCR primer ("GTGARTCATCGAATCTTTG").
pcr_primer_reverse (DNA-derived extension)	Reverse PCR primer ("TCCTCCGCTTATTGATATGC").
pcr_primer_name_forward (DNA-derived extension)	Name of the forward PCR primer ("Next-fITS7").
pcr_primer_name_reverse (DNA-derived extension)	Name of the reverse PCR primer ("Next-ITS4").
pcr_primer_reference (DNA-derived extension)	Reference for the primers (doi.org/10.1111/j.1574-6941.2012.01437.x).
env_broad_scale (DNA-derived extension)	The major environmental system using subclasses of ENVO’s biome class ("peatland").
lib_layout (DNA-derived extension)	The configuration of reads ("paired").
seq_meth (DNA-derived extension)	Sequencing method used ("Illumina MiSeq").
otu_class_appr (DNA-derived extension)	Approach/algorithm and clustering level ("Internal de-novo clustering with an identity parameter of 99% (QIIME vsearch cluster-features-de-novo)").
otu_seq_comp_appr (DNA-derived extension)	Tool and thresholds used to assign "species-level" names to OTUs ("Classification classify-sklearn (QIIME feature-classifier classify-sklearn) on a classifier that was trained using the naive Bayes classifier algorithm (QIIME feature-classifier fit-classifier-naive-bayes)").
otu_db (DNA-derived extension)	Reference database ("Clustering based on the UNITE database (version 9.0 16 October 2022) using cluster-features-closed-reference with 97% identity parameter (QIIME vsearch cluster-features-closed-reference)").
taxonRank (Occurrence core)	The taxonomic rank of the scientificName.
basisOfRecord (Occurrence core)	The specific nature of the data record ("materialSample").

### Data set 2.

#### Data set name

Metadata of sampling strategy during metabarcoding analysis (Zenodo archive)

#### Data format

CSV

#### Character set

UTF-8

#### Download URL

https://doi.org/10.5281/zenodo.10259604

#### Description

The table presents supplementary materials and contains metadata on sampling strategy during metabarcoding analysis of four types of substrates in two ombrotrophic bog habitats, with other experimental and environmental parameters included in the analyses.

**Data set 2.** 

Column label	Column description
Sample_ID	A unique identifier of the sample.
eventDate	Date when the sampling of substrate was made.
Substrate	Substrate of the sample ("Mycorrhizal roots", "Plant litter", "Wooden dowels", "Peat").
Conservation	Conservation method of the sample ("Drying", "Freezing").
DNA_extraction_kit	DNA extraction kit ("SileksMagNA").
Sampling_approach	Sampling approach ("Composite from 5 points 5 m apart, totalling in 5 g of fresh weight", "Single sample from one point, 0.25 g of fresh weight").
Extraction_repica	Extraction replica, if existing.
Depth	Depth at which the sample was extracted (applied for "Peat" in "Substrate").
Vegetation	Vegetation type where the sample was extracted ("Graminoid-sphagnum lawn of ombrotrophic raised bog", "Treed pine-dwarfshrubs-sphagnum ombrotrophic raised bog").
Locality	Locality name where the sample was extracted ("Mukhrino field station, Mukhrino Bog, 60.88909°N, 68.70244°E", "Shapsha field station, Chistoe Bog, 61.06551°N, 69.45863°E".
Plot_number	Plot number where the sample was extracted.
Plant_host	Plant host from which plant litter or mycorrhizal roots were extracted (totally 16 plant hosts).
Number_of_reads	Total number of reads in this sample.

## Additional information

### Conclusions

**Figure 9.**
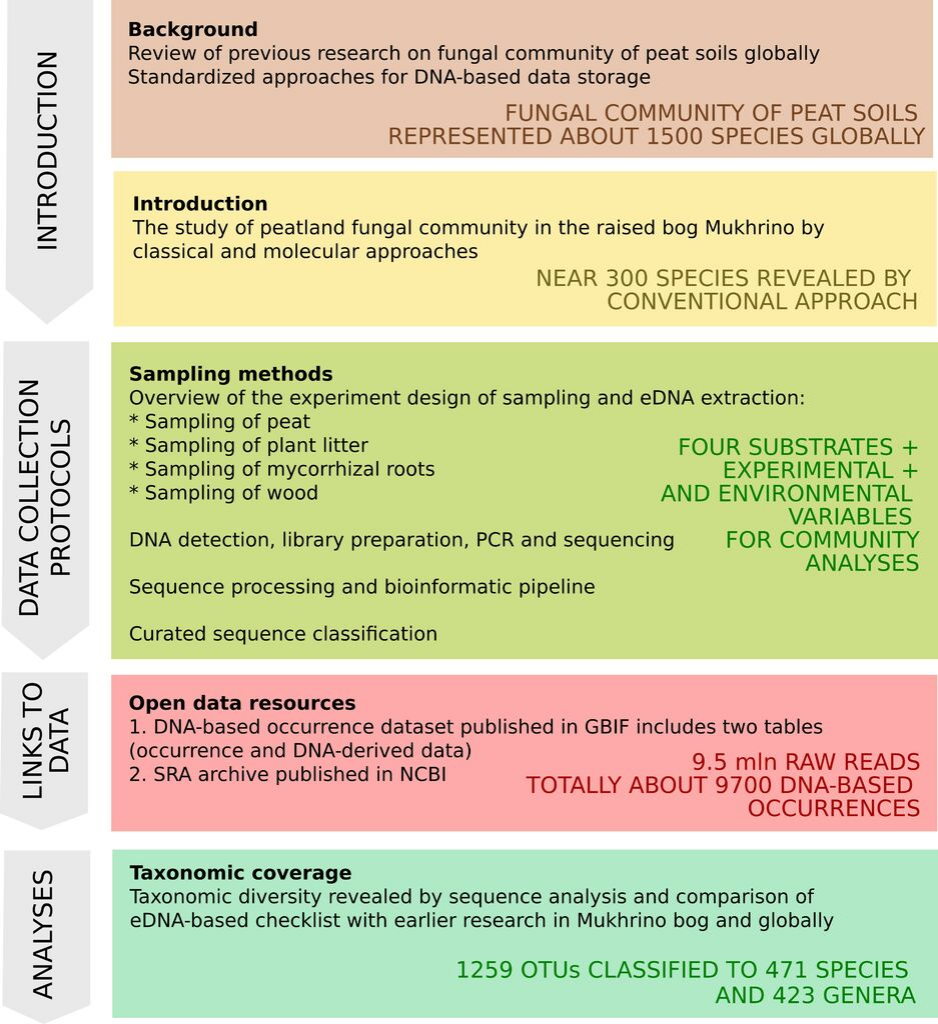
The general representation of the data paper structure and results.

The paper presents metabarcoding data on fungal communities of peat and other substrates sampled in the raised bog Mukhrino in north-western Siberia. Two datasets were published in open source depositories: a DNA-derived occurrence dataset published in GBIF and sequence reads archive of raw FastQ files published in NCBI. The methods of experiment design, sampling, bioinformatic piplines and data resources are described in detail. The layout of the data paper is resresented in Fig. 9.

## Figures and Tables

**Table 1.** Taxonomic structure of fungal community revealed by sequence analysis of eDNA in Mukhrino Bog.

	**Kingdom**	**Phyla**	**Class**	**Order**	**Family**	**Genus**	**Species**
Total number of taxa	1	7	30	86	223	423	471
Number of OTUs identified to this level	32	67	83	152	131	267	527
Percentage of OTUs identified to this level	3%	5%	7%	12%	10%	21%	42%

**Table 2.** Curated sequence classification results and comparison with machine identification at species level

**ID**	**Machine identification**	**Curated identification**	**Curation type**
1	*Cortinariuspluvius* (Fr.) Fr. 1838	*Cortinarius* sp. 9	reduced to genus level
2	*Lepiotaneophana* Morgan	*Lepiota* sp.	
3	*Mycenasemivestipes* (Peck) A.H. Sm. 1947	*Mycena* sp. 2	
4	*Tomentellalongisterigmata* X. Lu, K. Steffen & H.S. Yuan 2018	*Tomentella* sp.	
5	*Bovistapromontorii* Kreisel 1967	*Bovistaaestivalis* (Bonord.) Demoulin 1979	re-identified to other species
7	*Cortinariushydrotelamonioides* Rob. Henry 1970	*Cortinariuskauffmanianus* A.H. Sm.	
8	*Cortinariuspaleaceus* Fr. 1838	*Cortinariuslindstroemii* Niskanen, Kytov. & Liimat. 2020	
9	*Flammulaabieticola* (A.H. Sm. & Hesler) E.J. Tian & Matheny 2020	*Flammulaalnicola* (Fr.) P. Kumm. 1871	
10	*Galerinacalyptrata* P.D. Orton 1960	Galerinacf.calyptrata P.D. Orton 1960	
11	*Inocybetigrina* R. Heim 1931	*Inocybeflocculosa* Sacc. 1887	
12	*Lentinellusflabelliformis* (Bolton) S. Ito 1959	*Lentinellusmicheneri* Lentinellusmicheneri	
13	*Suillussubluteus* (Peck) Snell 1944	*Suilluspraetermissus* Zvyagina & Svetash. 2021	
14	*Lactarius* Pers. 1797	*Lactariustabidus* Fr. 1838	improved identification to species level
15	*Cortinarius* (Pers.) Gray 1821	*Cortinariusbataillei* (J. Favre ex M.M. Moser) Høil. 1984	
16	*Cortinarius* (Pers.) Gray 1821	*Cortinariusglandicolor* (Fr.) Fr. 1838	
17	*Cortinarius* (Pers.) Gray 1821	*Cortinariuskauffmanianus* A.H. Sm. 1933	
18	*Cortinarius* (Pers.) Gray 1821	*Cortinariuslindstroemii* Niskanen, Kytov. & Liimat. 2020	
19	*Cortinarius* (Pers.) Gray 1821	*Cortinariusominosus* Bidaud 1994	
20	*Cortinarius* (Pers.) Gray 1821	*Cortinariustenuifulvescens* Kytöv., Niskanen & Liimat. 2016	
21	*Cortinarius* (Pers.) Gray 1821	*Cortinariusbicolor* Cooke 1887	
22	*Cortinarius* (Pers.) Gray 1821	*Cortinariuscollinitus* (Sowerby) Gray 1821	
23	*Hypholoma* (Fr.) P. Kumm. 1871	*Bogbodiauda* (Pers.) Redhead 2013	
24	*Lycoperdon* Pers. 1794	*Lycoperdonperlatum* Pers. 1796	
25	Cortinariaceae	*Cortinariusglandicolor* (Fr.) Fr. 1838	
26	Cortinariaceae	*Cortinariuscollinitus* (Sowerby) Gray 1821	
27	Cortinariaceae	*Thaxterogastercausticus* (Fr.) Niskanen & Liimat. 2022	

## References

[B10625535] Abarenkov K., Andersson A. F., Bissett A., Finstad A. G., Fossøy F., Grosjean M., Hope M., Jeppesen T. S., Kõljalg U., Lundin D., Nilsson R. N., Prager M., Provoost D., Schigel D., Suominen S., Svenningsen C., Frøslev T. G. Publishing DNA-derived data through biodiversity data platforms, v1.3. Copenhagen: GBIF Secretariat.

[B10625007] Artz Rebekka R. E. (2013). Microbial Community Structure and Carbon Substrate use in Northern Peatlands. Carbon Cycling in Northern Peatlands.

[B10625425] Elliott David R., Caporn Simon J. M., Nwaishi Felix, Nilsson R. Henrik, Sen Robin (2015). Bacterial and Fungal Communities in a Degraded Ombrotrophic Peatland Undergoing Natural and Managed Re-Vegetation. PLOS ONE.

[B10625886] Filippova Nina (2012). Discomycetes from plant, leave and sphagnum litter in ombrotrophic bog (West Siberia). Environmental Dynamics and Global Climate Change.

[B10625895] Filippova Nina, Zmitrovich Ivan (2013). Wood decay community of raised bogs in West Siberia. Environmental Dynamics and Global Climate Change.

[B10625153] Filippova Nina, Lapshina Elena (2019). Sampling event dataset on five-year observations of macrofungi fruit bodies in raised bogs, Western Siberia, Russia. Biodiversity Data Journal.

[B11069747] Filippova N. (2023). Metadata of sampling strategy during metabarcoding analysis (Journal publication supplement) [Data set].

[B10625108] Filippova Nina, Rudykina Elena (2023). Literature occurrence database of global fungal diversity in peatlands. Version 1.7. Yugra State University Biological Collection (YSU BC). Occurrence dataset.

[B10625116] Filippova Nina, Rudykina Elena, Zvyagina Elena (2023). The checklist of macrofungi of raised bogs: barcoding of accumulated collection following the 9-year plot-based monitoring in Northwestern Siberia. Version 1.3. Yugra State University Biological Collection (YSU BC). Occurrence dataset.

[B10625137] Filippova Nina, Zvyagina Elena, Ishmanov Tagir (2023). DNA-based occurrence dataset on peatland fungal communities studied by metabarcoding in Northwestern Siberia. Version 1.6. Yugra State University Biological Collection (YSU BC). Occurrence dataset.

[B10625145] Filippova Nina, Rudykina Elena, Dobrynina Alevtina, Zvyagina Elena (2023). Plot-based observations of macrofungi in raised bogs in Western Siberia (2014-2022). Version 1.39. Yugra State University Biological Collection (YSU BC). Sampling event dataset.

[B10625034] Filippova Nina, Zvyagina Elena, Rudykina Elena, Dobrynina Alevtina, Bolshakov Sergey (2023). The diversity of macromycetes in peatlands: nine years of plot-based monitoring and barcoding in the raised bog "Mukhrino", West Siberia. Biodiversity Data Journal.

[B10625904] Filippova N. V. (2015). On the communities of fungi of raised bogs in taiga belt of West Siberia. II. Microfungi on plant litter. Mycology and phytopathology.

[B10625877] Filippova N. V., Thormann M. N. (2015). The fungal consortium of Andromedapolifolia in bog habitats. Mires and Peat.

[B10625435] Garcés-Pastor Sandra, Wangensteen Owen S., Pérez-Haase Aaron, Pèlachs Albert, Pérez-Obiol Ramon, Cañellas-Boltà Núria, Mariani Stefano, Vegas-Vilarrúbia Teresa (2019). DNA metabarcoding reveals modern and past eukaryotic communities in a high-mountain peat bog system. Journal of Paleolimnology.

[B10625407] Hibbett David S., Ohman Anders, Kirk Paul M. (2009). Fungal ecology catches fire. New Phytologist.

[B11297897] Ihrmark Katarina, Bödeker Inga T M, Cruz-Martinez Karelyn, Friberg Hanna, Kubartova Ariana, Schenck Jessica, Strid Ylva, Stenlid Jan, Brandström-Durling Mikael, Clemmensen Karina E, Lindahl Björn D (2012). New primers to amplify the fungal ITS2 region--evaluation by 454-sequencing of artificial and natural communities.. FEMS microbiology ecology.

[B10625416] Jackson Colin R., Liew Kong Cheng, Yule Catherine M. (2008). Structural and Functional Changes with Depth in Microbial Communities in a Tropical Malaysian Peat Swamp Forest. Microbial Ecology.

[B10625913] Kachalkin A. V. (2010). Дрожжевые сообщества сфагновых мхов.

[B10625316] Kirpotin Sergey N., Antoshkina Olga A., Berezin Alexandr E., Elshehawi Samer, Feurdean Angelica, Lapshina Elena D., Pokrovsky Oleg S., Peregon Anna M., Semenova Natalia M., Tanneberger Franziska, Volkov Igor V., Volkova Irina I., Joosten Hans (2021). Great Vasyugan Mire: How the world’s largest peatland helps addressing the world’s largest problems. Ambio.

[B10625334] Kremenetski K. V, Velichko A. A, Borisova O. K, MacDonald G. M, Smith L. C, Frey K. E, Orlova L. A (2003). Peatlands of the Western Siberian lowlands: current knowledge on zonation, carbon content and Late Quaternary history. Quaternary Science Reviews.

[B10625921] Lapshina Elena Dmitrievna, Koneva Vera Anatol'evna (2010). Species diversity of ground lichens in the raised bog vegetationof the Irtysh left-bank terraces. Environmental Dynamics and Global Climate Change.

[B10791384] Liimatainen Kare, Niskanen Tuula, Dima Bálint, Ammirati Joseph F., Kirk Paul M., Kytövuori Ilkka (2020). Mission impossible completed: unlocking the nomenclature of the largest and most complicated subgenus of Cortinarius, Telamonia. Fungal Diversity.

[B10625494] Martorelli Irene, Helwerda Leon S., Kerkvliet Jesse, Gomes Sofia I. F., Nuytinck Jorinde, van der Werff Chivany R. A., Ramackers Guus J., Gultyaev Alexander P., Merckx Vincent S. F. T., Verbeek Fons J. (2020). Fungal metabarcoding data integration framework for the MycoDiversity DataBase (MDDB). Journal of Integrative Bioinformatics.

[B10625465] Nilsson R. Henrik, Anslan Sten, Bahram Mohammad, Wurzbacher Christian, Baldrian Petr, Tedersoo Leho (2018). Mycobiome diversity: high-throughput sequencing and identification of fungi. Nature Reviews Microbiology.

[B10625476] Nilsson Rolf Henrik, Larsson Karl-Henrik, Taylor Andy F S, Bengtsson-Palme Johan, Jeppesen Thomas S, Schigel Dmitry, Kennedy Peter, Picard Kathryn, Glöckner Frank Oliver, Tedersoo Leho, Saar Irja, Kõljalg Urmas, Abarenkov Kessy (2018). The UNITE database for molecular identification of fungi: handling dark taxa and parallel taxonomic classifications. Nucleic Acids Research.

[B10625025] Rydin Håkan, Jeglum John K. (2006). Diversity of life in peatlands. The Biology of Peatlands.

[B10623403] Shumskaya Maria, Lorusso Nicholas, Patel Urvi, Leigh Madison, Somervuo Panu, Schigel Dmitry (2023). ﻿MycoPins: a metabarcoding-based method to monitor fungal colonization of fine woody debris. MycoKeys.

[B10623416] Tedersoo Leho, Bahram Mohammad, Zinger Lucie, Nilsson R. Henrik, Kennedy Peter G., Yang Teng, Anslan Sten, Mikryukov Vladimir (2022). Best practices in metabarcoding of fungi: From experimental design to results. Molecular Ecology.

[B10625016] Thormann Markus N. (2006). Diversity and function of fungi in peatlands: A carbon cycling perspective. Canadian Journal of Soil Science.

[B10625360] Thormann M. N., Rice A. V. (2007). Fungi from peatlands. Fungal Diversity.

[B10625448] Vašutová Martina, Jiroušek Martin, Hájek Michal (2021). High fungal substrate specificity limits the utility of environmental DNA to detect fungal diversity in bogs. Ecological Indicators.

[B10625369] Větrovský Tomáš, Morais Daniel, Kohout Petr, Lepinay Clémentine, Algora Camelia, Awokunle Hollá Sandra, Bahnmann Barbara Doreen, Bílohnědá Květa, Brabcová Vendula, D’Alò Federica, Human Zander Rainier, Jomura Mayuko, Kolařík Miroslav, Kvasničková Jana, Lladó Salvador, López-Mondéjar Rubén, Martinović Tijana, Mašínová Tereza, Meszárošová Lenka, Michalčíková Lenka, Michalová Tereza, Mundra Sunil, Navrátilová Diana, Odriozola Iñaki, Piché-Choquette Sarah, Štursová Martina, Švec Karel, Tláskal Vojtěch, Urbanová Michaela, Vlk Lukáš, Voříšková Jana, Žifčáková Lucia, Baldrian Petr (2020). GlobalFungi, a global database of fungal occurrences from high-throughput-sequencing metabarcoding studies. Scientific Data.

[B11297875] White T. J., Bruns T., Lee S., Taylor J. W., Innis M. A., Gelfand D. H., Sninsky J. J., White T. J. (1990). PCR Protocols: A Guide to Methods and Applications.

[B10625297] Wider R. K., Vitt D. H. (2006). Boreal Peatland Ecosystems.

[B10625307] Wieder R. Kelman, Vitt Dale H., Benscoter Brian W. (2006). Peatlands and the Boreal Forest. Ecological Studies.

